# Portable near-infrared spectroscopy and linear support vector machine classifiers for rapid screening of subclinical inflammation in dairy goat milk

**DOI:** 10.3168/jdsc.2026-1021

**Published:** 2026-06-17

**Authors:** Francesco Tangorra, Elena Ighina, Eleonora Buoio, Federica Santandrea, Martina Penati, Sara Fusar Poli, Matteo Mezzetti, Maria Filippa Addis

**Affiliations:** 1 Department of Veterinary Medicine and Animal Science, Lodi Campus, University of Milan, 26900 Lodi, Italy; 2 Department of Animal Sciences, Food and Nutrition (DIANA), Università Cattolica del Sacro Cuore, 29122 Piacenza, Italy; 3 Laboratorio di Malattie Infettive degli Animali (MiLab), University of Milan, 26900 Lodi, Italy

## Abstract

We evaluated the applicability of portable near-infrared spectroscopy (NIRS) to the rapid screening of goat milk for alterations related to mammary inflammation using the mastitis marker cathelicidin (Cath) as an inflammation-related reference outcome and SCC as the conventional routine reference. Diffuse-reflectance spectra (900–1,700 nm) were acquired directly through milk tubes (n = 228), preprocessed by standard normal variate (SNV), and reduced to 5 chemically interpretable spectral windows. An unsupervised principal component analysis was computed on the full SNV preprocessed spectral dataset, and samples were assigned to training (n = 171) and independent validation (n = 57) sets by principal component 1-quantile stratified randomization. Linear-kernel support vector machine (SVM) classifiers were trained to predict Cath status (positive defined as the presence of a reactive band at 15 kDa in the lane corresponding to the sample) and SCC class (positive defined as SCC >1,000,000 cells/mL). On validation, NIRS demonstrated associations with both reference outcomes. The Cath status model and the SCC class model yielded sensitivity of 0.69 versus 0.52, specificity of 0.90 versus 0.72, and area under the receiver operating characteristics curve of 0.80 versus 0.62, respectively. This was in line with the biological reliability of Cath as an inflammation marker, although paired bootstrap comparisons did not show statistically significant differences on the restricted validation set. Overall, portable NIRS combined with SVM showed preliminary promise as a rapid, nondestructive field triage approach for screening milk alterations associated with mammary inflammation in dairy goats.

## Graphical Abstract

**Figure:**
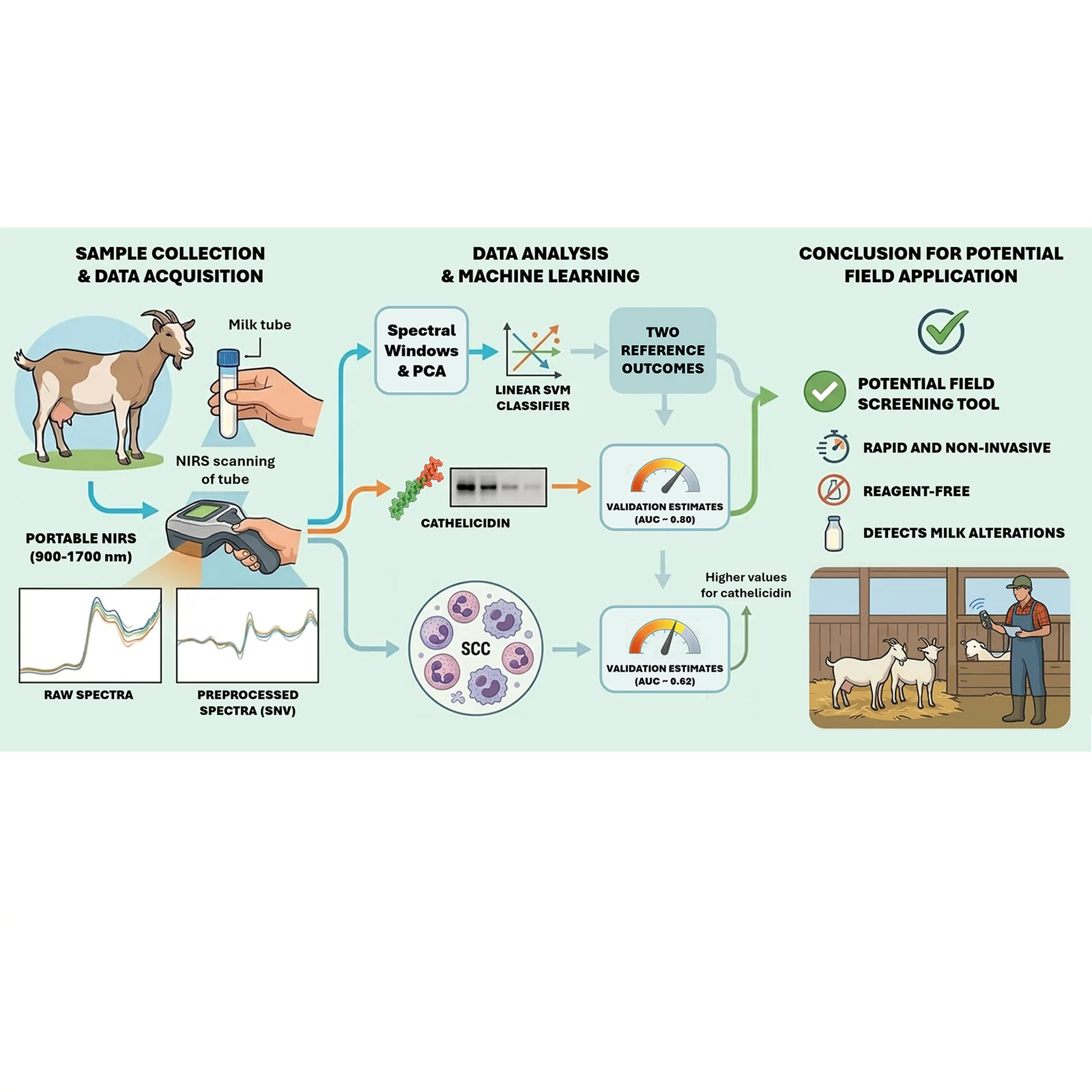

Mastitis is one of the most economically impacting diseases in dairy production due to the connected losses in both milk quality and quantity across all dairy species (Ruegg, 2017), and it is mainly caused by an IMI. In goats, IMI are due to a complex and diverse nature of etiological agents, predominantly non-*aureus* staphylococci and mammaliicocci (**NASM**), with *Staphylococcus caprae* as the most frequently isolated species (Bernier Gosselin et al., 2020). Major pathogens such as *Staphylococcus aureus*, *Mannheimia haemolytica*, and *Serratia* spp. can cause acute clinical mastitis. However, chronic subclinical infections by minor pathogens are far more prevalent in goat herds, making the detection of subclinical infections even more critical for herd health management and milk quality assurance (Koop et al., 2010; Mavrogianni et al., 2011; Tibebu et al., 2025). Traditional diagnostic approaches rely on laboratory-based SCC and rapid on-farm test methods such as the California Mastitis Test (**CMT**), although both have limitations in goats. The SCC physiological values are higher and more physiologically variable than in cattle, and thresholds vary significantly throughout lactation (Moroni et al., 2005; Souza et al., 2012). Furthermore, the impact on udder health by different NASM differs considerably among ruminant species, creating additional challenges in translating research findings across species (Bernier Gosselin et al., 2019; Rosa et al., 2022; Addis et al., 2024). Rapid on-farm alternatives to laboratory-based udder health assessment include the CMT and milk electrical conductivity measurements, which can also be integrated into automated milking systems. However, these approaches have important limitations: CMT is operator-dependent and semiquantitative, whereas conductivity measurements, although objective, may have limited or inconsistent diagnostic value in goats, where conductivity changes associated with mammary inflammation are generally less pronounced than in cattle (McDougall et al., 2001).

Alternative biomarkers have been extensively investigated to overcome these limitations. Among the biomarkers investigated for mastitis detection, cathelicidins (**Cath**) are of particular interest because they are innate immune antimicrobial peptides that increase markedly in milk during intramammary infection and inflammation (Zanetti, 2005; Addis et al., 2017). Unlike SCC, which in goats is influenced by physiological factors such as stage of lactation and apocrine milk secretion, Cath is more specifically associated with infection-driven mammary inflammation and has shown a closer relationship with udder inflammatory status in dairy ruminants (Giagu et al., 2022). However, laboratory methods for measuring inflammatory protein markers remain less suitable for rapid on-farm decision-making.

Near-infrared spectroscopy (**NIRS**) is a rapid, nondestructive technique increasingly available in handheld formats suitable for field and real-time measurements. In dairy science, beyond routine milk composition analysis (Tangorra et al., 2024), NIRS has also shown promise for detecting subclinical mastitis in cattle through chemometric classification approaches (da Silva Pereira et al., 2024; Rodríguez-Hernández et al., 2025). In NIRS, the partial least squares (**PLS**) approach is one of the most widely used chemometric approaches because it can effectively handle highly collinear spectral predictors. For classification tasks, commonly used methods include PLS-discriminant analysis (DA) and soft independent modeling of class analogy (SIMCA). As with any supervised chemometric approach, robust model development requires a sufficiently large and representative dataset that captures the variability expected in unknown samples. With the increasing availability of portable near-infrared devices, alternative supervised learning methods such as support vector machine (**SVM**) have also attracted interest for classification tasks. Its main advantages are lower computational cost and the ability to achieve good performance with fewer training samples than many other machine-learning classifiers (Cervantes et al., 2020).

Building on these considerations, this study evaluated portable NIRS combined with SVM as a tool for detecting inflammatory changes in milk composition, with the aim of enabling rapid field assessment of udder inflammation. Accordingly, 2 reference outcomes were considered: Cath status, as a biomarker more specifically linked to mammary inflammation in goats, and SCC, as the conventional routine reference.

Milk samples were obtained from 2 commercial dairy farms (farm 1 and farm 2) located in northern Italy and breeding Saanen and Alpine goats, respectively. The sampling and analytical unit was the half-udder milk sample. Thus, the dataset included 228 half-udder milk samples from 114 goats (25 from farm 1 and 89 from farm 2) at 239 ± 12 DIM. This approach was used because each gland half can have a distinct infection and inflammatory status. However, we cannot exclude the possibility that inflammation or infection in one half-udder may influence SCC, Cath abundance, or milk composition in the contralateral half through systemic or local physiological responses. Therefore, although each half-udder was analyzed as an individual mammary unit, potential within-goat clustering and contralateral effects were not explicitly modeled and should be considered a limitation of the present study. Milk was collected before the morning milking into sterile polypropylene screw-cap 15-mL tubes. Before milk collection, teats were cleaned and disinfected with chlorhexidine-embedded, single-use towels, and the foremilk was discarded. Goats showing visible signs of clinical mastitis or milk alterations were excluded from the study. The samples were immediately transported to the laboratory in refrigerated containers. Ethical approval was not required because milk samples were collected as part of routine herd udder health monitoring, before the morning milking, and no additional animal handling or invasive procedures were performed specifically for research purposes.

Somatic cell counting was carried out with a Bentley Somacount 150 (Bentley Instruments, USA), whereas the Cath status was determined by western blot as previously described (Pisanu et al., 2020) with minor modifications. Briefly, 5 µL of milk was diluted in 45 µL of loading buffer (Sigma-Aldrich, St. Louis, MO), denatured at 95°C for 3 min, and centrifuged at 9,400 × *g* for 1 min at room temperature. Ten microliters of sample was separated by SDS-PAGE on precast Mini-PROTEAN TGX Gels (Bio-Rad Laboratories, Hercules, CA) and transferred to nitrocellulose using a Trans-Blot Turbo (Bio-Rad). Membranes were blocked with EveryBlot Blocking Buffer (Bio-Rad) and incubated with anti-Cath mAb at 1 µg/mL (Addis et al., 2016), followed by horseradish peroxidase-conjugated anti-mouse secondary antibody (Thermo Fisher Scientific, Waltham, MA). Reactive bands were detected using Clarity Western ECL Substrate (Bio-Rad) and acquired with an IBright 1500 instrument (Thermo Fisher Scientific). Positive and negative controls were included in all gels. Cathelicidin-positive or -negative status was defined as the presence or absence, respectively, of a reactive band at 15 kDa in the lane corresponding to the sample. The assay was used here to assign binary Cath status and not to quantify protein concentration; therefore, a minimum detectable concentration was not determined in the present study.

The bacteriological analysis of milk was performed in line with standard procedures (Adkins et al., 2017). Briefly, 10 µL of half-udder milk was plated onto Columbia agar and incubated overnight at 37°C. After visual inspection, visible colonies were subjected to species identification, and plates were incubated for an additional day when needed. Because 10 µL of milk was plated, bacterial growth was interpreted semiquantitatively from colony counts on the plate, with 1 colony corresponding approximately to 100 cfu/mL and 10 colonies to 1,000 cfu/mL. Accordingly, milk was classified as positive at thresholds of ≥100 cfu/mL for *S. aureus* and ≥1,000 cfu/mL for other pathogens (colonies of similar morphology), negative when no relevant growth was observed, and contaminated when more than 2 colony types were present. Bacteria were identified by MALDI-TOF MS with a Bruker MBT platform (Bruker Daltonics, Bremen, Germany) as described previously (Rosa et al., 2022).

A handheld near-infrared spectrometer (DLP NIRscan Nano DMD, Texas Instruments, USA) was used to collect diffuse-reflectance spectra from goat milk samples directly through 15-mL polypropylene screw-cap tubes over 900–1,700 nm (10-nm resolution). To maximize practical applicability, milk was scanned directly in the standard laboratory sampling tubes without chemical or physical pretreatment, mirroring a workflow that could be readily integrated into routine herd health monitoring. The instrument was warmed up for 10 min and a background spectrum was recorded using a Spectralon standard (99% reflectance) before the first measurement. The optical spot (10 × 10 mm) was placed in direct contact with the tube wall. Each sample (half-udder milk considered an independent physiological unit) was scanned at 3 positions by rotating the device on the tube wall and 3 replicate spectra were averaged to yield 1 representative spectrum per sample. A total of 228 mean spectra were available. Spectra were preprocessed using standard normal variate (**SNV**) to reduce multiplicative scatter effects before multivariate analysis (Barnes et al., 1989). The SNV was selected following internal comparisons with Savitzky–Golay first- and second-derivative preprocessing, using the standard settings implemented in JMP (Worley and Ash, 2024).

To reduce dimensionality while preserving chemical interpretability, model development was restricted to 5 spectral windows associated with major milk absorptions, using all wavelength points within each window as predictors: 920–950 nm (C–H, lipids), 960–990 nm (O–H, water), 1,180–1,230 nm (C–H, lipids), 1,420–1,490 nm (O–H, water), and 1,500–1,620 nm (N–H, proteins). An unsupervised principal component analysis was computed on the full SNV preprocessed spectral dataset and samples were randomly assigned to the training (75%; n = 171) and independent validation (25%; n = 57) sets using stratification by principal component 1 (**PC1**) score quantiles to preserve the distribution of spectral variability. Two separate linear-kernel SVM classifiers (JMP Pro 19, SAS Institute Inc., Cary, NC) were trained using the same predictors but different reference outcomes: (1) Cath status (positive or negative, laboratory assay) as the primary inflammation-related outcome, and (2) SCC class (possible issue or healthy), with possible issue operationally defined as SCC >1,000,000 cells/mL based on a literature-derived pragmatic threshold (Contreras et al., 2007; Koop et al., 2011; Moroni et al., 2005). The final prediction formulas were applied unchanged to the independent validation set. Predicted classes were assigned using a fixed probability threshold of 0.50 for both outcomes. Model performance was evaluated on the validation set using confusion matrices, sensitivity, specificity, accuracy, *F*_1_ score, Matthews correlation coefficient (**MCC**), and area under the receiver operating characteristic curve (**AUC**). Differences in sensitivity, specificity, and MCC between the Cath status and SCC class validation analyses were assessed using paired bootstrap resampling of the independent validation set with 10,000 resamples in JMP Pro 19. In each bootstrap replicate, the 57 validation samples were resampled with replacement, performance metrics were recalculated for both analyses, and the Cath minus SCC difference was recorded. Percentile 95% bootstrap confidence intervals were obtained from the 2.5th and 97.5th percentiles of the bootstrap distributions. Differences were considered statistically supported when the 95% bootstrap confidence interval did not include zero.

Based on the measurements made on 228 half-udder samples, the mean ± SD SCC value was 1,007 ± 935 cells/mL, with a median value of 795 (range 1–5,265). Cathelicidin-positive samples were 80 (35.1%). The number of samples positive to bacteriological culture was 38 (16.7%), with the following identifications: *S. caprae* and *S. equorum* (9 each), *S. lentus* (5), *Serratia* spp. and *S. aureus* (3 each), *S. gallinarum*, *S. simulans*, *S. xylosus* (2 each), *Aerococcus viridans*, *Corynebacterium stationis*, and *S. chromogenes* (1 each).

The mean SNV spectra for Cath-positive and Cath-negative samples are shown in Figure 1. The 2 groups were broadly similar across 900 to 1,700 nm, with only subtle differences in regions associated with lipid (C–H), water (O–H), and protein (N–H) absorptions (Yang et al., 2022). An unsupervised principal component analysis score plot (PC1 vs. principal component 2 [**PC2**]) of the full SNV preprocessed spectral dataset is shown in Figure 2. The PC1 and PC2 explained 83.3% and 9.47% of the total variance, respectively. Cathelicidin-positive and -negative samples showed substantial overlap in the PC1–PC2 space, indicating no clear unsupervised group separation in the first 2 principal components.

**Figure 1:**
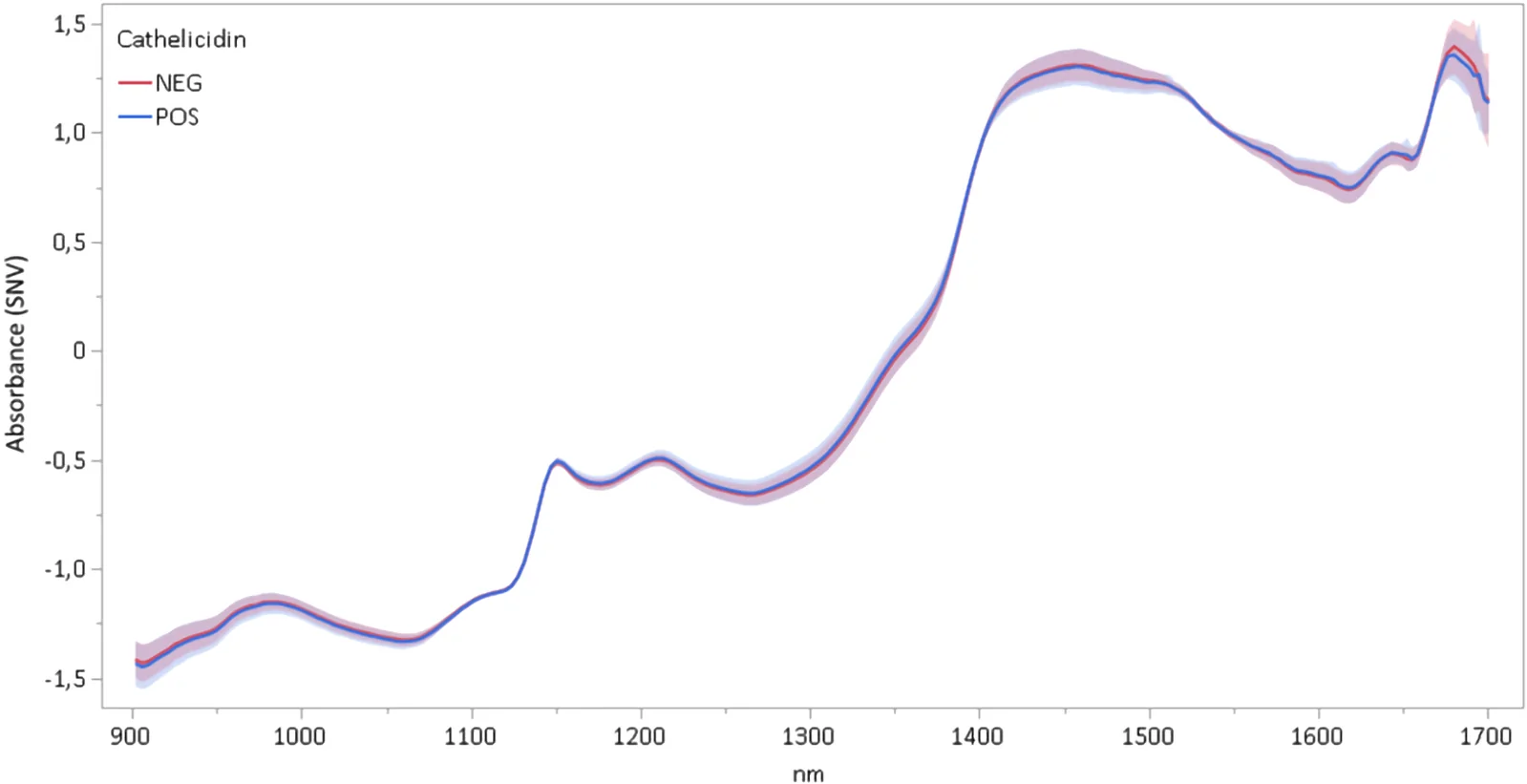
Mean SNV-preprocessed spectra of cathelicidin-negative (NEG) and cathelicidin-positive (POS) milk samples across 900 to 1,700 nm. Shaded areas represent mean ± 1 SD for each group.

**Figure 2:**
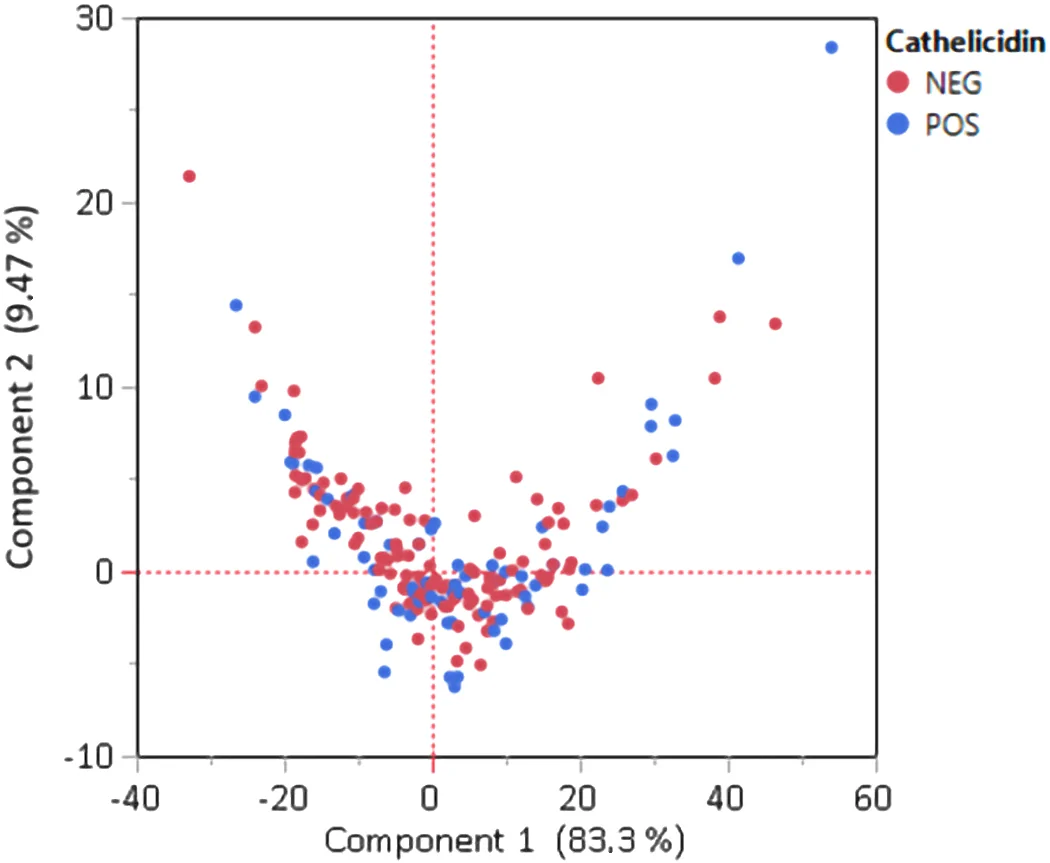
Score plot of the unsupervised principal component analysis computed on the full SNV preprocessed spectral dataset. Samples are colored according to cathelicidin status (negative [NEG] or positive [POS]).

Using the 5 chemically interpretable windows as predictors, the 2 linear SVM models were evaluated on the independent validation set (n = 57). The cost values were 2.14 for the Cath model and 2.59 for the SCC model, indicating similar regularization strength in the 2 linear SVM classifiers. For Cath, the validation confusion matrix was true positives (**TP**) = 11, false negatives (**FN**) = 5, false positives (**FP**) = 4, and true negatives (**TN**) = 37, corresponding to sensitivity = 0.69, specificity = 0.90, accuracy = 0.84, *F*_1_ = 0.71, MCC = 0.60, and AUC = 0.80. For SCC class, the validation confusion matrix was TP = 11, FN = 10, FP = 10, and TN = 26, corresponding to sensitivity = 0.52, specificity = 0.72, accuracy = 0.65, *F*_1_ = 0.52, MCC = 0.25, and AUC = 0.62. Differences in validation performance between the Cath status and SCC class analyses were formally assessed by paired bootstrap resampling for sensitivity, specificity, and MCC. The 95% bootstrap confidence intervals for Cath minus SCC were −0.317 to 0.292 for sensitivity, −0.042 to 0.335 for specificity, and −0.211 to 0.516 for MCC. Because all intervals included zero, the validation data did not provide statistical evidence of differences between the 2 analyses for these metrics (Table 1).

**Table 1:** Performance of SVM classifiers for predicting Cath status and SCC class in the external validation set (n = 57)

Outcome^1^	N	TP	FN	FP	TN	Sensitivity	Specificity	Accuracy	AUC	*F*_1_	MCC
Cathelicidin status	57	11	5	4	37	0.69	0.90	0.84	0.80	0.71	0.60
SCC class	57	11	10	10	26	0.52	0.72	0.65	0.62	0.52	0.25

1 Cathelicidin status (positive or negative) was determined by western immunoblotting. SCC (possible issue) was defined as SCC >1,000,000 cells/mL. TP = true positives; FN = false negatives, FP = false positives; TN = true negatives. AUC = area under the receiver operating characteristic curve; *F*_1_ = *F*_1_ score; MCC = Matthews correlation coefficient.

A robust biological foundation is critical for training accurate chemometric algorithms in dairy ruminant inflammation in general, and in dairy goats in particular. Somatic cell count is currently considered the standard indicator for mastitis in dairy animals, but its reliability in goats is frequently confounded by noninfectious or noninflammatory factors (Souza et al., 2012). Consequently, SCC may represent a noisy reference standard for chemometric modeling, whereas Cath may provide a biologically relevant reference because it is an immune response protein upregulated during mammary inflammation (Zanetti, 2005; Giagu et al., 2022). Overall, the NIRS models successfully captured spectral variations linked to both reference outcomes. The numerically higher point estimates obtained with the Cath-status model are in line with Cath being a specific biological marker for mammary inflammation in goats, but paired bootstrap comparisons did not provide statistical evidence of differences between the 2 parameters. The practical value of the Cath-based model for screening depends not only on sensitivity and specificity but also on disease prevalence. Assuming the Cath prevalence observed in the present dataset (35.1%), the estimated PPV and NPV were 0.79 and 0.84, respectively. Therefore, the model's moderate sensitivity remains an important limitation because some inflamed half-udders may still be classified as negative. Taken together, these results suggest that the near-infrared spectra contained information related to the reference outcomes used in this study. Previous literature supports milk Cath as a biologically relevant marker of mammary inflammation in dairy ruminants, including goats (Giagu et al., 2022). However, our NIRS results should not be interpreted as showing that the spectral signal directly measures inflammation or Cath. Rather, they show that milk spectral variation was associated with milk alterations co-occurring with those affecting the reference classifications under the conditions of this study. These associations should be interpreted cautiously, as neither reference outcome can be assumed to represent a perfect standard for mammary inflammation, and pathogen-specific aspects may also influence milk biomarker responses and spectral variation (Addis et al., 2017). A further limitation is that spectra were acquired directly through polypropylene tubes. Although this choice was made to maximize practical applicability and was kept constant for all samples, polypropylene is not spectrally inert in the near-infrared region and may have contributed to the measured signal. Because this contribution was not independently assessed using empty-tube controls or alternative containers, its influence on model performance cannot be fully excluded.

From a practical perspective, this method may offer useful advantages as a rapid screening approach. The NIRS-based workflow can provide results within seconds and may help identify milk samples showing spectral or compositional alterations associated with inflammation-related reference outcomes. This could support on-farm triage and the selection of animals or samples requiring follow-up testing. However, several limitations should be considered. In goats, mammary involution processes can also lead to immune system activation and increased Cath levels (Sun et al., 2024), so positive results in late lactation should be interpreted with caution. Additionally, the current model cannot confirm IMI or identify the etiological agent. Bacteriological culture remains necessary for that purpose. In conclusion, portable NIRS combined with linear SVM showed preliminary feasibility for rapid, nondestructive screening of milk alterations associated with mammary inflammation in dairy goats. Both the Cath-status and SCC-class models yielded encouraging validation estimates, demonstrating the versatility of NIRS. The numerically higher estimates obtained with Cath further support the biological relevance as an inflammation marker, although statistical differences between the 2 models were not observed in this dataset. Therefore, the approach should be considered exploratory and requires confirmation in larger, independent datasets before implementation as an on-farm decision-support tool.
